# Strategien zur Eindämmung von Antibiotikaresistenzen in der Humanmedizin aus Sicht des Bundesministeriums für Gesundheit

**DOI:** 10.1007/s00103-025-04049-x

**Published:** 2025-05-05

**Authors:** Alexandra Clarici

**Affiliations:** Referat 632 „One Health, Antimikrobielle Resistenzen“, Bundesministerium für Gesundheit, Mauerstraße 29, 10117 Berlin, Deutschland

**Keywords:** Deutsche Antibiotika-Resistenzstrategie, One Health, Prävention, Sachgerechter Antibiotikaeinsatz, Surveillance, German Antimicrobial Resistance Strategy, One Health, Prevention, Appropriate use of antibiotics, Surveillance

## Abstract

Antibiotikaresistenzen (AMR) nehmen weltweit zu. Die komplexen Zusammenhänge bei deren Entstehung und Verbreitung fordern ein Maßnahmenbündel sowie ein sektorübergreifendes Vorgehen unter Berücksichtigung des One-Health-Ansatzes. Die Deutsche Antibiotika-Resistenzstrategie „DART“ fasst die Maßnahmen für Deutschland zusammen. Aufbauend auf 2 Vorgängerstrategien beschreibt die im Jahr 2023 verabschiedete „DART 2030“ die innerhalb der Laufzeit zu erreichenden strategischen Ziele. Sie wird ergänzt durch einen im Mai 2024 veröffentlichten Aktionsplan, der konkrete Aktivitäten in den 6 Handlungsfeldern enthält: Prävention, Surveillance und Monitoring, sachgerechter Antibiotikaeinsatz, inklusive Labordiagnostik, Kommunikation und Kooperation, europäische und internationale Zusammenarbeit sowie Forschung und Entwicklung. Eine wichtige Aktion in der Humanmedizin ist die Sicherstellung der Verfügbarkeit von Empfehlungen/Leitlinien zur Infektionsprävention sowie zu Diagnostik und Therapie von Infektionen, insbesondere solchen, bei denen Resistenzen eine Rolle spielen. Der Aufbau infektiologischer Expertise soll unterstützt und die Surveillance von Resistenzen und des Antibiotikaverbrauchs auch sektorübergreifend gestärkt werden. Bewährte Austauschformate werden fortgeführt, um die Kommunikation innerhalb und zwischen den Sektoren zu verbessern. In der Bevölkerung soll das Wissen über Infektionen, deren Behandlung und den adäquaten Einsatz von Antibiotika verbessert werden.

Der Aktionsplan soll regelmäßig überprüft und aktualisiert werden. Dies wird mit einer Berichterstattung verbunden, die auch auf die Fortschritte bei der Erreichung von konkreten Zielvorgaben für die Humanmedizin eingehen wird.

## Abstract (en)

Antibiotic resistance (AMR) is increasing worldwide. The complex interrelationships in their development and spread require a bundle of measures and a cross-sectoral approach that considers the One Health approach. The German Antibiotic Resistance Strategy “DART” summarizes the measures for Germany. Building on two predecessor strategies, “DART 2030,” which was adopted in 2023, describes the strategic goals to be achieved during its term. It is supplemented by an action plan published in May 2024, which contains specific activities in the six fields of action: prevention, surveillance and monitoring, appropriate use of antibiotics including laboratory diagnostics, communication and cooperation, European and international cooperation, and research and development. Important actions in human medicine include ensuring the availability of recommendations/guidelines on infection prevention and on the diagnosis and treatment of infections, particularly those in which resistance plays a role. The development of expertise in infectiology will be supported and the surveillance of resistance and antibiotic consumption strengthened across all sectors. Proven exchange formats will be continued in order to improve communication within and between sectors. The population’s knowledge about infections, their treatment, and the appropriate use of antibiotics will be strengthened.

The action plan will be regularly reviewed and updated. This will be linked to reporting, which will also address progress in achieving specific targets for human medicine.

## Einleitung

Antibiotikaresistenzen (AMR)^1^ nehmen weltweit zu und stellen eine immer größer werdende Herausforderung bei der Versorgung von Patientinnen und Patienten dar. Für Deutschland gehen Berechnungen, die anlässlich der deutschen G7-Präsidentschaft im Jahr 2022 erfolgten, für das Jahr 2019 von 45.700 Todesfällen im Zusammenhang mit resistenten Erregern und 9650 Todesfällen direkt durch AMR aus [1]. AMR bewirken, dass bakterielle Infektionen schwerer zu behandeln sind. Die Folge sind längere und schwerere Krankheitsverläufe und mehr Todesfälle. Zudem ist die moderne Medizin z. B. bei Gelenksersatz, Chemotherapie bei Krebserkrankungen oder der Versorgung Frühgeborener auf wirksame Antibiotika angewiesen.

Die Entwicklung von AMR ist ein natürlicher Vorgang, der durch einen unsachgemäßen Gebrauch von Antibiotika beschleunigt wird. Hygienemängel tragen dazu bei, dass sich Erreger, auch solche mit Resistenzen, ausbreiten können. Resistente Bakterien können u. a. durch direkten Kontakt oder über Lebensmittel übertragen werden und über Ausscheidungen in die Umwelt gelangen. Antibiotika, die z. B. über Abwässer in die Umwelt gelangen, können ebenfalls zur Resistenzproblematik beitragen. Das macht deutlich, dass AMR nur mit einem sektorenübergreifenden „One-Health-Ansatz“ und durch gemeinsame Anstrengungen auf regionaler, nationaler und internationaler Ebene entgegnet werden kann. Allerdings unterscheiden sich die Rahmenbedingungen in den einzelnen Ländern z. T. erheblich, weswegen auf nationale Strukturen zugeschnittene Lösungen gefunden werden müssen.

Im Jahr 2015 hat die Weltgesundheitsversammlung den Globalen Aktionsplan (GAP) zu AMR verabschiedet [2]. Er fordert die Mitgliedstaaten auf, nationale, dem One-Health-Ansatz folgende Aktionspläne zu entwickeln und Maßnahmen umzusetzen.

Die in Deutschland zu ergreifenden Maßnahmen werden in der Deutschen Antibiotika-Resistenzstrategie (DART) zusammengefasst. Nachdem die erste Strategie „DART“ bereits im Jahr 2008 veröffentlicht wurde, folgte 2015 kurz nach Verabschiedung des GAP die Nachfolgestrategie „DART 2020“, die die Handlungsempfehlungen des GAP aufgriff. Beide Strategien wurden unter der Federführung des Bundesministeriums für Gesundheit (BMG) gemeinsam mit den Bundesministerien für Ernährung und Landwirtschaft (BMEL) sowie für Bildung und Forschung (BMBF) erarbeitet. Die Fortschritte bei ihrer Umsetzung wurden in einem Abschlussbericht dargestellt [3].

Mit den beiden Strategien wurden wichtige Strukturen etabliert und weiterentwickelt, die auch weiterhin die Basis für die Aktivitäten in Deutschland sind. Das beinhaltet im Humanbereich beispielsweise den Auf- und Ausbau der Surveillance-Systeme für AMR und den Antibiotikaverbrauch am Robert Koch-Institut (RKI), die Etablierung der „Kommission Antiinfektiva, Resistenz und Therapie“ (ART), die wie auch die „Kommission für Infektionsprävention in medizinischen Einrichtungen und in Einrichtungen und Unternehmen der Pflege und Eingliederungshilfe“ (KRINKO) im Infektionsschutzgesetz (IfSG) verankert ist und deren Empfehlungen verpflichtenden Charakter haben, sowie die Etablierung regionaler MRE-Netzwerke (MRE = multiresistente Erreger) in vielen Regionen Deutschlands. Über das IfSG sind zudem die Leiter medizinischer Einrichtungen dazu verpflichtet, Daten zu AMR und zum Antibiotikaverbrauch aufzuzeichnen und regelmäßig zu bewerten.

Im europäischen Vergleich sind die Resistenzraten in Deutschland eher niedrig. Das ist auch auf die erfolgreiche Umsetzung der bisherigen Maßnahmen zurückzuführen. Diese müssen jedoch fortgeführt und angepasst werden, um den Status quo dauerhaft halten bzw. die Resistenzlage verbessern zu können. Zudem müssen Entwicklungen, wie z. B. das Auftreten neuer Resistenzmechanismen und deren Verbreitung über Ländergrenzen hinweg, berücksichtigt werden.

Aufbauend auf den Erkenntnissen aus der Vorgängerstrategie, wurde im Jahr 2023 die aktuelle Strategie „DART 2030“ entwickelt [4]. Nachdem den zentralen Akteuren Gelegenheit zur Kommentierung gegeben wurde, wurde sie im April 2023 vom Bundeskabinett verabschiedet. Mit den Beiträgen der Bundesministerien für Umwelt, Naturschutz, nukleare Sicherheit und Verbraucherschutz (BMUV) sowie für wirtschaftliche Zusammenarbeit und Entwicklung (BMZ) wurde die nationale AMR-Strategie gegenüber der Vorgängerstrategie um wichtige Inhalte ergänzt.

Um in der Laufzeit bis 2030 flexibler auf sich ändernde Rahmenbedingungen reagieren zu können, wurde zudem ein geändertes Format gewählt. Die DART 2030 beschreibt die bis zum Jahr 2030 zu erreichenden strategischen Ziele in 6 Handlungsfeldern. Sie wird ergänzt durch einen Aktionsplan mit konkreten Maßnahmen. Er soll regelmäßig aktualisiert werden. Der erste Aktionsplan wurde im Mai 2024 veröffentlicht und enthält Maßnahmen, die prioritär bis Ende des Jahres 2025 umgesetzt werden sollen [5]. Er adressiert auch Verpflichtungen, die die G7 unter der deutschen Präsidentschaft im Jahr 2022 und mit der Verabschiedung der „Empfehlung des Rates zur Intensivierung der EU-Maßnahmen zur Bekämpfung antimikrobieller Resistenz im Rahmen des Konzeptes Eine Gesundheit“ im Juni 2023 eingegangen ist. So wurden beispielsweise nationale Zielvorgaben für den Antibiotikaverbrauch und bestimmte Resistenzraten im Humanbereich festgelegt. Sie sollen dabei unterstützen, den Fortschritt bei der Umsetzung der Strategie zu messen.

Wichtige Maßnahmen werden in den Handlungsfeldern Prävention, Surveillance und Monitoring, sachgerechter Antibiotikaeinsatz, inklusive Labordiagnostik, Kommunikation und Kooperation sowie Forschung und Entwicklung umgesetzt.

## 1. Prävention

Die Prävention behandlungsbedürftiger Infektionskrankheiten trägt dazu bei, die Anwendung von Antibiotika und damit den auf die Bakterien wirkenden Selektionsdruck zu vermindern. Weniger Infektionen führen auch dazu, dass weniger Antibiotika und resistente Erreger in die Umwelt gelangen.

Die Maßnahmen zur Stärkung der Infektionsprävention adressieren sowohl die Bevölkerung, die das notwendige Wissen dafür haben muss, als auch die Fachöffentlichkeit. Für die Infektionsprävention im Zusammenhang mit medizinischen Maßnahmen stellen die KRINKO-Empfehlungen den Stand der medizinischen Wissenschaft dar und geben Orientierung. Sie werden regelmäßig überprüft und angepasst. Im September 2022 wurde das IfSG geändert und das Mandat der KRINKO auf die Bereiche Pflege und Eingliederungshilfe ausgeweitet. Diese sollen in künftigen Empfehlungen zur Infektionsprävention stärker berücksichtigt werden.

Empfehlungen und Leitlinien zur Hygiene mit dem Ziel der Infektionsprävention müssen in den Einrichtungen vor Ort zur Verfügung gestellt und angewendet werden. Implementierungshindernisse müssen erkannt und beseitigt werden. Dafür ist es wichtig, die Aufmerksamkeit für die Infektionsprävention sowie die daraus resultierende mögliche Kostenreduktion auf allen Ebenen in medizinischen Einrichtungen und Einrichtungen der Pflege zu verankern [4]. Nur so kann langfristig ein positiver Effekt auf die Häufigkeit der behandlungsbedürftigen Infektionen erwartet werden.

Eine wichtige und erfolgreiche Maßnahme der Infektionsprävention stellen Impfungen dar – auch gegen virale Infektionen, bei denen bakterielle Superinfektionen oftmals zu schweren Verläufen mit der Konsequenz einer Antibiotikatherapie führen. Gleichzeitig können Impfungen Fehlindikationen für Antibiotika im ambulanten Bereich reduzieren. Der Nationale Impfplan formuliert die für Deutschland angestrebten Impfquotenziele. Seine konsequente Umsetzung kann dazu beitragen, die Entstehung und Verbreitung von AMR zu reduzieren [4].

## 2. Surveillance und Monitoring

Daten zum Antibiotikaeinsatz und zu AMR werden u. a. für die Trenderkennung, für gezielte Maßnahmen und die Messung von deren Wirkung benötigt. In Deutschland werden Daten zu AMR in der Humanmedizin in der am RKI etablierten Antibiotika-Resistenz-Surveillance (ARS) zusammengeführt [4]. Neben dem Überblick über die AMR-Situation auf Bundesebene ermöglicht das System den teilnehmenden Laboren über Feedbackberichte einen Überblick über die lokale Resistenzlage und seltene Resistenzen. Für den Antibiotikaverbrauch im stationären Bereich steht u. a. die Antibiotika-Verbrauchs-Surveillance (AVS) am RKI zur Verfügung. Sie dient auch dazu, den stationären Bereich bei der Umsetzung der gesetzlichen Vorgaben aus dem IfSG zur Aufzeichnung und Bewertung von Daten zum Antibiotikaverbrauch zu unterstützen. Das System „Antibiotika-Resistenz und -Verbrauch – integrierte Analyse (ARVIA)“ zeigt mögliche Abhängigkeiten der Resistenzlage von Antibiotikaverordnungen und unterstützt lokale ABS-Aktivitäten. Um detailliertere Aussagen zum Vorkommen und zur Ausbreitung von AMR treffen zu können, sollen die nationalen Surveillance-Systeme weiter ausgebaut werden [4]. Dem Deutschen Elektronischen Melde- und Informationssystem für den Infektionsschutz (DEMIS) wird dabei eine zentrale Rolle zukommen. Insbesondere für die Reserveantibiotika gemäß § 35a Absatz 1c Fünftes Buch Sozialgesetzbuch (SGB V) ist es wichtig, einen Überblick über den Einsatz und die Resistenzentwicklung zu erhalten, um frühzeitig ungünstige Entwicklungen zu erkennen und Maßnahmen ergreifen zu können. Zudem wird die Ausweitung der AVS auf den gesamten ambulanten Versorgungssektor angestrebt, verbunden mit der Möglichkeit zum einrichtungsindividuellen Feedback. Ein im Projekt „Interventionen zum rationalen Einsatz von Antibiotika im ambulanten Bereich – Machbarkeitsstudie zur Surveillance (SAMBA)“ des RKI erprobtes System mit einem Feedbackmechanismus soll verstetigt werden. Feedbackmechanismen kommen im ambulanten Bereich eine wichtige Bedeutung zu, da sie den Vergleich des eigenen Verordnungsverhaltens mit dem anderer Vertreterinnen und Vertreter derselben Fachdisziplin erlauben.

Die nationalen Systeme dienen auch dazu, den Berichtspflichten gegenüber den europäischen Systemen „European Antimicrobial Resistance Surveillance Network“ (EARS-Net) und „European Surveillance of Antimicrobial Consumption Network“ (ESAC-Net) sowie dem „Global Antimicrobial Resistance and Use Surveillance-System“ (GLASS) nachkommen zu können. Sie tragen so dazu bei, auch die globale Datenlage zu verbessern.

Unter der deutschen G7-Präsidentschaft im Jahr 2022 haben sich die G7-Gesundheits- und Agrarministerinnen und -minister dazu verpflichtet, integrierte Surveillance-Systeme zu AMR und zum Antibiotikaeinsatz aus dem Human- und Veterinärbereich sowie der Landwirtschaft und der Umwelt auf- bzw. auszubauen [4]. Die integrierte Datenauswertung dient der übergreifenden Bewertung der Resistenzsituation in Deutschland. Molekularbiologische Untersuchungen von Erregern sind ein wichtiges Instrument, um z. B. Resistenzgene nachweisen und Infektionsketten nachverfolgen zu können. Daher soll auch die molekulare Surveillance von AMR weiter ausgebaut werden. Das betrifft auch die Abwasser-Surveillance für AMR. Langfristig können Sequenzdaten resistenter Erreger aus dem Humanbereich mit denen aus dem Veterinär- und Umweltbereich zusammengeführt werden, um sektorübergreifende Infektionsketten besser nachvollziehen und Aussagen zur Ausbreitung von Resistenzen über Sektorgrenzen hinweg treffen zu können. Die sektorübergreifende Auswertung von Daten aus den Surveillance- und Monitoringsystemen soll schrittweise ausgebaut werden [4].

## 3. Sachgerechter Antibiotikaeinsatz, inklusive Labordiagnostik

Antibiotic Stewardship (ABS) soll sowohl den indikationsgerechten Antibiotikaeinsatz als auch die bestmögliche antibiotische Behandlung sicherstellen und dazu beitragen, die zunehmende Resistenzentwicklung zu reduzieren oder zu vermeiden [4]. Wichtige Voraussetzung für ABS sind die Verfügbarkeit hochwertiger infektiologischer Leitlinien, ausreichend qualifiziertes Personal sowie Daten zu AMR und zum Antibiotikaverbrauch. Zur Stärkung des sachgerechten Antibiotikaeinsatzes wird an unterschiedlichen Stellen angesetzt.

Die Kommission ART beim RKI erstellt Empfehlungen mit allgemeinen Grundsätzen für Diagnostik und antimikrobielle Therapie, insbesondere bei Infektionen mit resistenten Krankheitserregern, und berichtet zu geeigneten Rahmenbedingungen einer sachgerechten antiinfektiven Therapie [5]. Sie kann zudem Prioritäten in der Erstellung oder Anpassung von Leitlinien festlegen. Die Fördermöglichkeiten über den Innovationsfonds beim Gemeinsamen Bundesausschuss sowie die BMG-Beauftragungen des Instituts für Qualität und Wirtschaftlichkeit im Gesundheitswesen (IQWiG) zu Evidenzrecherchen auf Vorschlag der Arbeitsgemeinschaft der Wissenschaftlichen Medizinischen Fachgesellschaften (AWMF) leisten einen wichtigen Beitrag dafür, dass aktuelle infektiologische S3-Leitlinien im AWMF-Leitlinienregister verfügbar sind [4]. Mit Förderung durch den Innovationsfonds konnten in den vergangenen Jahren bereits mehrere wichtige, infektiologische Krankheitsbilder betreffende S3-Leitlinien erarbeitet werden.

Auf dem 124. Ärztetag im Jahr 2021 wurde beschlossen, in Deutschland eine eigenständige Facharztweiterbildung „Innere Medizin und Infektiologie“ im Gebiet der inneren Medizin einzuführen. Um die Etablierung der neuen Facharztweiterbildung in der Anfangsphase zu unterstützen und die infektiologische Expertise in der Humanmedizin weiter zu stärken, ist seit Januar 2023 über das Krankenhausentgeltgesetz eine Förderung von Personaleinstellungen, Fort- und Weiterbildungsmaßnahmen sowie Beratungsleistungen in Infektiologie in stationären medizinischen Einrichtungen möglich. Das „Infektiologieförderprogramm“ ist aus dem Hygieneförderprogramm hervorgegangen, seine Laufzeit endet im Dezember 2025. Da sich das Programm ausschließlich an stationäre Einrichtungen wendet, infektiologische Expertise aber zunehmend auch im ambulanten Bereich benötigt wird, wird derzeit geprüft, wie dieser Bereich gestärkt werden kann [5]. Dem ambulanten Bereich kommt bei der Eindämmung von AMR eine besondere Rolle zu, da hier ca. 85 % der Antibiotika verordnet werden. Bei einem niedrigen Antibiotikaeinsatz werden in Deutschland vergleichsweise häufig Breitspektrumantibiotika verordnet. Zudem werden noch zu oft Antibiotika bei respiratorischen Virusinfekten eingesetzt. Strukturen und Maßnahmen müssen daher die spezifischen Erfordernisse dieses Bereichs berücksichtigen, um wirksam werden zu können.

Nicht zuletzt ist auch eine qualifizierte und technologisch adäquat ausgerüstete mikrobiologische Labordiagnostik eine wichtige Voraussetzung für ABS. Daher soll der Einsatz von Diagnostika, auch von Point-of-Care-Tests im ambulanten und stationären Bereich gestärkt und ausgebaut werden.

## 4. Kommunikation und Kooperation

Verschiedene Formate unterstützen den Austausch von Öffentlichkeit, Fachexpertinnen und -experten und politischen Entscheidungsträgern über die Sektoren hinweg.

Die Interministerielle Arbeitsgruppe Antibiotika-Resistenzen (IMAG AMR) dient der ressortübergreifenden Koordinierung, Anpassung und Erweiterung der nationalen AMR-Strategie. In ihr arbeiten seit dem Jahr 2008 die an der DART beteiligten Ministerien und Geschäftsbereichsbehörden zusammen. Zukünftig wird sich IMAG AMR noch stärker als bisher mit der Koordinierung von Maßnahmen und dem Monitoring der Umsetzung der DART 2030 befassen, um den im europäischen und globalen Rahmen gestiegenen Anforderungen nachzukommen.

In den vergangenen Jahren wurde immer deutlicher, wie komplex die Zusammenhänge bei der Resistenzausbreitung sind und dass jeder Sektor eine eigene Resistenzproblematik aufweist. Das Verhältnis der Sektoren untereinander ist jedoch weiterhin in vielen Fällen von unzureichendem Einblick in die Belange der jeweils anderen geprägt [4]. Im Jahr 2023 wurde daher ein Veranstaltungsformat initiiert, dass den aktiven Austausch zwischen Fachexpertinnen und -experten, zunächst nur aus der Human- und Veterinärmedizin, unterstützen soll und über das gemeinsame Interventionen angestoßen werden können. Der Workshop „Gemeinsam gegen Antimikrobielle Resistenzen: One Health-Dialog zwischen Mensch, Tier und Umwelt“ führte den Austausch im November 2024 unter Einbindung von Vertretern aus dem Umweltbereich und der Landwirtschaft weiter.

Unter Koordinierung des Öffentlichen Gesundheitsdienstes haben sich in vielen Regionen Deutschlands u. a. Krankenhäuser, Rehabilitationseinrichtungen, Pflegeheime und Arztpraxen in „regionalen MRE-Netzwerken“ zusammengeschlossen, um durch abgestimmte Maßnahmen die Weiterverbreitung von MRE einzudämmen. In regelmäßigen, vom RKI organisierten Treffen tauschen die Netzwerke ihre Erfahrungen aus. Die Zusammenarbeit von Ärztinnen und Ärzten in lokalen Netzwerken und Qualitätszirkeln sind eine wichtige Maßnahme, die den sachgerechten Antibiotikaeinsatz im ambulanten Bereich der Humanmedizin unterstützen kann. Zu diesem Schluss kamen mehrere Projekte, die mögliche Einflussfaktoren untersucht haben. Neben der Nutzung verschiedener Informationsformate zur Wissensvermittlung an Ärztinnen und Ärzte sowie Patientinnen und Patienten, einer integrierten Surveillance von Antibiotikaverbrauch und AMR, wie sie im System ARVIA erfolgt, regelmäßigen individuellen Feedbackberichten zu Antibiotikaverordnungen und der Stärkung der Arzt-Patienten-Kommunikation wurde auch die Verfügbarkeit von Kurzfassungen infektiologischer Leitlinien als wichtiger positiver Faktor genannt.

Über die derzeit mit BMG-Förderung laufende Kampagne #DeutschlandErkenntSepsis werden medizinisches Personal und Bevölkerung über die Prävention, Frühsymptome, Diagnose und Therapie einer Sepsis informiert. Über diese Kampagne hinaus sollen in der Bevölkerung auch die Kenntnisse über die Therapie von Infektionserkrankungen und den sachgerechten Einsatz von Antibiotika verbessert werden. Dabei geht es u. a. um den Zusammenhang von Infektionen und der Entwicklung einer Sepsis, der Bedeutung der Infektionsprävention durch Impfungen sowie Maßnahmen der Basishygiene. Die sich an die Bevölkerung richtenden Informationsangebote des Bundesinstituts für Öffentliche Gesundheit (BIÖG; ehemals Bundeszentrale für gesundheitliche Aufklärung) sollen fortgeführt und mit den fachlichen Informationsangeboten des RKI verknüpft werden. Bei der Wissensvermittlung soll noch stärker zielgruppenorientiert vorgegangen und die veränderte Mediennutzung infolge der Digitalisierung berücksichtigt werden.

## 5. Europäische und internationale Zusammenarbeit

AMR als globale Herausforderung kann nur in internationaler Zusammenarbeit bewältigt werden. Über den globalen Handel sowie Reisetätigkeiten können sich Resistenzen rasch weltweit ausbreiten. Dies macht deutlich, dass der Aufbau von Kapazitäten zur Eindämmung von AMR, die Stärkung von Infektionsprävention, Surveillance und Monitoring, der sachgerechte Einsatz von Antibiotika und Diagnostika sowie der Zugang zu Diagnostika und Antibiotika in anderen Ländern auch Deutschland zugutekommt.

Innerhalb der Europäischen Union (EU) erfolgt die Abstimmung der Mitgliedstaaten im One-Health-Netzwerk zu AMR. Sektorübergreifend werden aktuelle Entwicklungen, Herausforderungen und Lösungsansätze diskutiert und so die Umsetzung des im Jahr 2017 verabschiedeten „European One Health Action Plan against Antimicrobial Resistance“ unterstützt [6]. Dieser sieht u. a. vor, die EU zu einer Best-Practice-Region bei der Reduzierung von AMR zu machen.

Die Europäische Gemeinsame Aktion zu AMR und gesundheitsassoziierten Infektionen 2 (EU-JAMRAI 2) zielt darauf ab, die Mitgliedstaaten bei der Entwicklung und Umsetzung nationaler AMR-Aktionspläne konkret zu unterstützen, und fördert den Kapazitätsaufbau. Deutschland beteiligt sich an Arbeitspaketen zum sachgerechten Antibiotikaeinsatz, zur Infektionsprävention und -kontrolle und zu Surveillance sowie an Maßnahmen, die den Austausch zwischen den Mitgliedstaaten fördern und der Umsetzung und Weiterentwicklung der nationalen Aktionspläne dienen sollen.

Auf globaler Ebene wurden in den vergangenen Jahren in Ergänzung zu den bereits bestehenden Arbeitsgruppen und Projekten verschiedene, meist sektorübergreifende Gremien und Plattformen zu AMR etabliert bzw. befinden sich im Aufbau. Deutschland setzt sich zudem seit Langem dafür ein, dass die G7 und G20 AMR als ein prioritäres Thema behandeln, und hat es bei seinen eigenen Präsidentschaften auf die Agenda gesetzt. So sind die G7 im Jahr 2022 wichtige Verpflichtungen zu AMR und Sepsis eingegangen. Die Bundesregierung wird die Diskussionen in den internationalen Foren auch weiterhin aktiv vorantreiben und eng begleiten.

Im Jahr 2024 fanden mit dem High-Level-Meeting der Vereinten Nationen im September und der 4. Ministerkonferenz im November in Saudi-Arabien 2 hochrangige internationale Veranstaltungen zu AMR statt, von denen wichtige Impulse für die weltweite Reaktion auf AMR in den kommenden Jahren ausgehen. Zur Begleitung der internationalen Prozesse wurde im Februar 2024 am BMG die Position einer AMR-Botschafterin eingerichtet.

Das Meeting der Vereinten Nationen war nach 2016 das zweite hochrangige Treffen der UN zu AMR. Mit der Annahme der politischen Deklaration verpflichten sich die Mitgliedstaaten, u. a. nationale One-Health-AMR-Aktionspläne (NAP), inklusive nationaler Zielvorgaben zu AMR und Antibiotikaverbrauch, bis 2030 zu aktualisieren und zu implementieren sowie deren nachhaltige Finanzierung zu sichern. Der Fortschritt der Mitgliedstaaten bei der Umsetzung der NAP soll unter Nutzung bestehender Formate, wie z. B. der Multi-Stakeholder-Partnership-Plattform und der 2‑jährig stattfindenden Ministerkonferenzen, nachgehalten werden. Zudem soll der globale Zugang zu Antibiotika unter Berücksichtigung von deren sachgerechtem Einsatz sowie zu Diagnostika und Impfstoffen gestärkt werden.

## 6. Forschung und Entwicklung

Alle relevanten Forschungsbereiche, u. a. die Grundlagenforschung, die klinische Forschung, Versorgungsforschung, Forschung zu Public-Health-Fragen, Umwelt- und Klimaforschung und Kommunikation bis hin zu Forschung zum Zusammenhang der Gesundheit von Mensch, Tier und Umwelt, müssen gestärkt werden, um daraus gezielte Maßnahmen ableiten zu können. Dabei darf der nationale bzw. globale Forschungsbedarf nicht außer Acht gelassen werden.

Ein Forschungsschwerpunkt auf nationaler und internationaler Ebene wird auch künftig die Entwicklung neuer Antibiotika und anderer antimikrobiell wirkender Arzneimittel sein, die die insbesondere durch die Weltgesundheitsorganisation (WHO) priorisierten bakteriellen Erreger adressieren [4]. Abgesehen davon, dass die Entwicklung neuer Wirkstoffklassen immer aufwendiger wird, haben sich pharmazeutische Unternehmen aus der Erforschung und Entwicklung neuer antimikrobieller Wirkstoffe zurückgezogen, da die erwarteten Gewinne für Antibiotika deutlich geringer sind, als dies z. B. im Bereich der chronischen Erkrankungen oder der Onkologie der Fall ist. Antibiotika sollten möglichst selten eingesetzt werden, um ihre Wirkung zu erhalten. Diskutiert werden daher Anreizmechanismen, mit denen das Entkoppeln von Profit und Volumen gelingen kann. Ziel ist, die Pipeline mit innovativen Antibiotikakandidaten zu füllen.

Neben der Förderung von Forschung und Entwicklung neuer Antibiotika über „Push Incentives“ sind dafür auch „Pull Incentives“ erforderlich, die das teilweise Marktversagen ausgleichen. Auf nationaler Ebene hat Deutschland bereits im Jahr 2020 mit dem Gesetz für einen fairen Kassenwettbewerb in der gesetzlichen Krankenversicherung (GKV-FKG) einen Pull-Anreiz für innovative Antibiotika im deutschen Erstattungssystem eingeführt: Neuen Reserveantibiotika wird nunmehr im Nutzenbewertungsverfahren ein Sonderstatus zugesprochen. Der Zusatznutzen gilt für Reserveantibiotika als belegt. Die Neuregelung zielt explizit auf Reserveantibiotika ab. Hierunter werden insbesondere Antibiotika verstanden, die gegen die von der WHO priorisierten MRE wirken. Die gesetzgeberische Intention war, pharmazeutischen Unternehmern für Reserveantibiotika ein Preisniveau zu ermöglichen, das einen finanziellen Anreiz für Forschung und Entwicklung darstellt.

Die Entwicklung und Vermarktung von Antibiotika ist jedoch ein globaler Prozess, sodass Lösungen auf internationaler Ebene gefunden werden müssen. Die auch von Deutschland unterstützten internationalen Initiativen „Global Antibiotic Research and Development Partnership“ (GARDP) und „Combatting Antibiotic-Resistant Bacteria Pharmaceutical Accelerator“ (CARB-X) stärken komplementär die Entwicklung neuer Antibiotika von der frühen präklinischen Forschung bis hin zur klinischen Entwicklung, Zulassung und Zurverfügungstellung.

Das BMG setzt sich zudem seit Jahren in den internationalen Gremien, u. a. den G7 und G20, dafür ein, dass die Forschung und Entwicklung neuer Antibiotika dort diskutiert werden und ein Pull-Anreiz aus EU-Mitteln realisiert wird. Die „Health Emergency Preparedness and Response Authority“ (HERA) arbeitet derzeit an einem EU-finanzierten Pilotprojekt, um pharmazeutischen Unternehmern durch eine Umsatzgarantie einen Anreiz zur Markteinführung ihrer zugelassenen Antibiotika in den teilnehmenden Mitgliedstaaten zu bieten. Deutschland unterstützt das Vorhaben.

## Fazit

Die DART 2030 und der zugehörige Aktionsplan fassen zusammen, in welchen Bereichen innerhalb der Laufzeit bis 2030 angesetzt werden soll und welche Maßnahmen prioritär bis Ende 2025 umgesetzt werden sollen. Bei der anschließenden Überprüfung und Anpassung des Aktionsplans wird auch geprüft, inwiefern Anforderungen, die sich aus den internationalen Prozessen für Deutschland ergeben, in die nationale Strategie einfließen sollten.

Der Aktionsplan enthält bis 2030 zu erreichende Zielvorgaben für den Humanbereich, insbesondere zu Antibiotikaresistenzen und -verbrauch. Sie ergeben sich aus der im Juni 2023 verabschiedeten „Empfehlung des Rates zur Intensivierung der EU-Maßnahmen zur Bekämpfung antimikrobieller Resistenz im Rahmen des Konzepts Eine Gesundheit“ [7]. Die Ratsempfehlungen sehen ein Reduktionsziel für die EU vor, zu dessen Erreichen jeder Mitgliedstaat seinen Beitrag leisten muss. Dafür wurden für jeden Mitgliedstaat konkrete Vorschläge erarbeitet. Für den Aktionsplan wurden diese Ziele um 2 weitere Reduktionsziele – Senkung der sepsisbedingten Todesfälle und Senkung der Inzidenz von Blutstrominfektionen durch Vancomycin-resistente Enterokokken – ergänzt, die spezifisch deutsche Problembereiche adressieren. Die Berichterstattung wird auch auf den Fortschritt bei der Erreichung dieser Ziele eingehen [4].

Mit Maßnahmen in den 6 Handlungsfeldern der DART 2030 soll unter Berücksichtigung des One-Health-Ansatzes das übergeordnete gemeinsame Ziel, AMR zu vermeiden, erreicht werden. Die hier beschriebenen Maßnahmen, die im Humanbereich ansetzen bzw. die sektorübergreifend umgesetzt werden, werden ergänzt durch Aktivitäten, die die Veterinärmedizin, Landwirtschaft und Umwelt adressieren. Denn nur eine Kombination der verschiedenen Maßnahmen, die Zusammenarbeit auf allen Ebenen sowie faktenbasiertes Wissen bei den Akteurinnen und Akteuren und in der Bevölkerung werden langfristig zum Erfolg führen.

## Footnotes

1 Es wird die im internationalen Gebrauch übliche Abkürzung „AMR“ (für „antimikrobielle Resistenz“) verwendet, Ziel der DART 2030 ist jedoch die Reduktion von Antibiotikaresistenzen bei bakteriellen Erregern.

## References

[1] IHME, University of Washington (2022) Antimikrobielle Resistenzen: Krankheitslast in G7-Staaten und weltweit Ein dringender Aufruf zum Handeln. https://www.bundesgesundheitsministerium.de/fileadmin/Dateien/3_Downloads/G/G7/G7_AMR_de_2022.pdf. Zugegriffen: 9. Dez. 2024

[2] World Health Organization (2015) Global Action Plan on Antimicrobial Resistance. https://iris.who.int/bitstream/handle/10665/193736/9789241509763_eng.pdf?sequence=1. Zugegriffen: 9. Dez. 2024

[3] Bundesministerium für Gesundheit (2022) DART 2020 Abschlussbericht. https://www.bundesgesundheitsministerium.de/fileadmin/Dateien/3_Downloads/D/DART_2020/BMG_DART_2020_Abschlussbericht_bf.pdf. Zugegriffen: 9. Dez. 2024

[4] Bundesministerium für Gesundheit (2023) DART 2030 Deutsche Antibiotika-Resistenzstrategie. https://www.bundesgesundheitsministerium.de/fileadmin/Dateien/3_Downloads/A/Antibiotika-Resistenz-Strategie/DART_2030_bf.pdf. Zugegriffen: 9. Dez. 2024

[5] Bundesministerium für Gesundheit (2030) Aktionsplan zur DART. https://www.bundesgesundheitsministerium.de/fileadmin/Dateien/3_Downloads/A/Antibiotika-Resistenz-Strategie/240510_1._Aktionsplan_zur_DART_2030.pdf. Zugegriffen: 9. Dez. 2024

[6] European Commission (2017) A European One Health Action Plan against Antimicrobial Resistance (AMR). https://health.ec.europa.eu/document/download/353f40d1-f114-4c41-9755-c7e3f1da5378_de?filename=amr_2017_action-plan.pdf. Zugegriffen: 9. Dez. 2024

[7] Rat der Europäischen Union (2023) Empfehlung des Rates zur Intensivierung der EU-Maßnahmen zur Bekämpfung antimikrobieller Resistenz im Rahmen des Konzepts „Eine Gesundheit“. https://data.consilium.europa.eu/doc/document/ST-9581-2023-INIT/de/pdf. Zugegriffen: 9. Dez. 2024

